# Encapsulation of Olive Leaf Polyphenol-Rich Extract in Polymeric Micelles to Improve Its Intestinal Permeability

**DOI:** 10.3390/nano13243147

**Published:** 2023-12-15

**Authors:** Maria Camilla Bergonzi, Chiara De Stefani, Marzia Vasarri, Emilija Ivanova Stojcheva, Alba María Ramos-Pineda, Francesco Baldi, Anna Rita Bilia, Donatella Degl’Innocenti

**Affiliations:** 1 Department of Chemistry Ugo Schiff, University of Florence, Via Ugo Schiff 6, 50019 Sesto Fiorentino, Florence, Italy; chiara.destefani@unifi.it (C.D.S.); marzia.vasarri@unifi.it (M.V.); francesco.baldi1@edu.unifi.it (F.B.); ar.bilia@unifi.it (A.R.B.); 2 Department of Experimental and Clinical Biomedical Sciences “Mario Serio”, Viale Morgagni 50, 50134 Florence, Italy; donatella.deglinnocenti@unifi.it; 3 Natac Biotech SL, Electronica 7, 28923 Alcorcón, Spain; eivanova@natacgroup.com (E.I.S.); aramos@natacgroup.com (A.M.R.-P.)

**Keywords:** olive leaf, polyphenolic extract, oleuropein, polymeric micelles, Pluronics, TPGS, PAMPA, Caco-2, stability, permeability

## Abstract

In the present study, polymeric micelles were developed to improve the intestinal permeability of an extract of *Olea europaea* L. leaf with a high content of total polyphenols (49% *w*/*w*), with 41% *w*/*w* corresponding to the oleuropein amount. A pre-formulation study was conducted to obtain a stable formulation with a high loading capacity for extract. The freeze-drying process was considered to improve the stability of the formulation during storage. Micelles were characterized in terms of physical and chemical properties, encapsulation efficiency, stability, and in vitro release. The optimized system consisted of 15 mg/mL of extract, 20 mg/mL of Pluronic L121, 20 mg/mL of Pluronic F68, and 10 mg/mL of D-α-tocopheryl polyethylene glycol succinate (TPGS), with dimensions of 14.21 ± 0.14 nm, a polydisersity index (PdI) of 0.19 ± 0.05 and an encapsulation efficiency of 66.21 ± 1.11%. The influence of the micelles on polyphenol permeability was evaluated using both Parallel Artificial Membrane Permeability Assay (PAMPA) and the Caco-2 cell monolayer. In both assays, the polymeric micelles improved the permeation of polyphenols, as demonstrated by the increase in P_e_ and P_app_ values.

## 1. Introduction

*Olea europaea* L. is one of the most valuable fruit trees producing olive oil, which is important for human nutrition. In addition, it is a plant rich in bioactive secondary metabolites belonging to different chemical classes and identified in different parts of the tree, e.g., in the bark, root, wood, and leaf [1]. Olive plants are rich in phenolic compounds and, in *Olea europaea* L., oleuropein, demethyl-oleuropein, ligstroside, and oleoside represent the predominant phenolic oleosides. Oleuropein (OLE) and its derivatives, such as tyrosol and hydroxytyrosol, are among the major components of olive leaves, and are also present in the oil and fruits [2]. Olive leaves are used in traditional medicine as a natural remedy. Therefore, promoting the recovery of generally wasted products, such as leaves, is an innovative intervention in the circular and sustainable bio-economy. In fact, there is a need to consider the growing demand for natural compounds that can be used for human health and well-being while respecting the environment. Olive leaf by-products are rich in bioactive compounds which exhibit various nutritional and pharmacological properties, although their application is limited by poor water solubility and bioavailability.

The extraction of polyphenol-rich fraction from the waste material is important in terms of environmental impact. Every year, 4.5 million tons of olive leaves are produced worldwide by the olive oil industry and they represent a problem, because they must be removed from the fields and mills. However, olive leaves have great potential to be transformed into by-products, due to their high OLE content. Furthermore, the application of pharmaceutical formulations to improve the biopharmaceutical characteristics of the active compounds in olive leaves represents a comprehensive exploitation of this biomass for food, cosmetic, or pharmaceutical applications.

The polyphenols have different beneficial effects, as they are antioxidant, anti-inflammatory, anticancer, hypoglycemic, and neuroprotective [3,4]. OLE has been widely described in the literature for its multiple bioactive properties related to its antioxidant and free scavenger activities [5]. Many of the pharmacological activities of OLE have been attributed to its potent antioxidant efficacy, such as hypoglycemic, antiviral, antimicrobial, platelet antiaggregant, anticancer, hypolipidemic, and anti-inflammatory activities [6,7,8,9]. Recently, OLE has also been recognized as a molecule that can modulate certain receptors [10,11] and affect epigenetics [12]. Although OLE has been shown to be useful in the prevention or treatment of a large number of diseases and pathological conditions [13], its therapeutic use is limited by the poor stability to light, and sensitivity to oxygen, temperature, pH, and enzymes [14,15]. In addition, OLE has low permeability and undergoes a complex biotransformation process during gastric digestion. Taken together, these instability phenomena result in the compromised bioactivity and bioavailability of OLE. In the present research, novel polymeric micelles were developed to improve the intestinal permeability of a phenolic extract of *Olea europaea* L. leaf (OPA40) with high content of total polyphenols (49% *w*/*w*), with 41% *w*/*w* corresponding to OLE.

The use of nanomaterials for efficient and intelligent drug delivery is a goal for improving and achieving specific therapeutic effects for human health. The technique of encapsulation of hydrophobic and hydrophilic natural compounds allows them to be protected from chemical and enzymatic degradation in the gastrointestinal tract and, as a result, to increase their permeability to the intestinal barrier. Therefore, in recent decades, nanotechnology has become increasingly important in improving the solubility, bioavailability, permeability, and stability of natural compounds and extracts [16,17,18,19]. The formulation of the extracts into dosage forms is a complex process, because an extract is a mixture of chemicals and related compounds that contribute to the pharmacological activity. Polymeric micelles are nanosized systems, composed of amphiphilic polymers, that self-assemble over a critical micellar concentration (CMC), producing colloid aggregates with a hydrophobic core and a hydrophilic shell that can also be functionalized. Polymeric micelles are capable in increasing solubility, stability [20,21] and provide many in vivo advantages; they improve the mean residence time of a drug in the bloodstream, increase the bioavailability, overcome low drug permeability across the biological barriers and allow for the reduction of per os doses and possible related toxicity [22,23]. They can be prepared more easily, at a cheaper cost, and with an easier scale-up, compared to other nanocarriers [24].

In this work, a novel mixed micelle was developed to deliver OPA40. Indeed, OLE, the main polyphenol of the extract, is a hydrophilic molecule with a relatively high molecular weight. These factors reduce its permeability through the intestinal mucosa [25]. After a pre-formulation study, the best formulation was characterized in terms of physical and chemical properties, encapsulation efficiency, stability during storage, and drug release. The influence of the micelles on the polyphenols’ permeability was also evaluated in vitro, employing both artificial membrane (PAMPA assay) [26,27] and the Caco-2 cell monolayer [28,29].

## 2. Materials and Methods

### 2.1. Materials and Chemicals

Polyphenol-rich extract (OPA40) was provided by Natac Biotech SL (Alcorón, Spain). We also utilized Pluronic F68, Pluronic L121, D-a-tocopheryl polyethylene glycol succinate (TPGS), phosphate-buffered saline BioPerformance Certified pH 7.4 (PBS), Tween 80, lecithin (≥99%, TLC) lyophilized powder, cholesterol BioReagent (≥99%); all analytical grade and HPLC-grade solvents were provided by Sigma Aldrich Italia (Milan, Italy). Distilled water was obtained from a Simplicity UV Water Purification System from Merck Millipore (Darmstadt, Germany). The PAMPA filter plate (pore size 0.45 μm) was purchased from Millipore Corporation (Tullagreen, Carrigtwohill, Cork, Ireland). Oleuropein, hydroxytyrosol, verbascoside, and luteolin were from Sigma-Aldrich, Darmstadt, Germany; luteolin-7-O-glucoside and apigenin-7-O-glucoside were from Extrasynthese, Genay, France.

For cell culture, DMEM (Dulbecco’s modified Eagle’s medium), FBS (fetal bovine serum), penicillin and streptomycin, L-glutamine, trypsin–EDTA solution, phosphate Buffered saline (PBS), 1-(4,5-dimethylthiazol-2-yl)-3,5-diphenyl formazan (MTT) were purchased from Merck KGaA (Darmstadt, DA, Germany). Sterile single-use plastic was purchased from Sarsdedt (Milan, Italy). For the permeability assay, Lucifer Yellow (LY), Hanks’ balanced salt solution (HBSS), and HEPES solution were purchased from Merck KGaA (Darmstadt, DA, Germany).

### 2.2. Olea europaea L. Extract (OPA40) Preparation

Leaves from *Olea europaea* L. were harvested in May of 2022. After picking, the olive leaves were sun-dried until the required moisture content was reached. The initial moisture content of the olive leaves was 47.72% (*w*/*w*). Before extraction, the leaves were ground to small pieces with 250–500 µm particle size. The extraction was performed with a hydroalcoholic solution, with a plant/solvent ratio of 1:20. Then, the extract was concentrated and fractionated by column chromatography using a commercial resin. The fraction enriched in oleuropein (41.67% oleuropein *w*/*w*) was brought to dryness under reduced pressure at 40 °C and homogenized by milling. The final extract (OPA40) was characterized by HPLC-DAD and HPLC-MS/MS analyses, as previously reported [30,31,32,33]. Determination of the polyphenols was carried out at 233 nm, 280 nm, 330 nm, and 355 nm using oleuropein, hydroxytyrosol, verbascoside, luteolin, luteolin-7-O-glucoside, and apegienin-7-O-glucoside as internal standards [34].

### 2.3. Chromatography Conditions

The HPLC system consisted of a 1200 high performance liquid chromatograph (HPLC) equipped with a diode array detector (DAD) from Agilent Spa (Rome, Italy). The column was a C18 Luna Omega Polar (150 × 4.6, 3 µm, Agilent Technology, Santa Clara, CA, USA). The analytical method was selected from Cecchi et al. [35]. An OLE calibration curve was constructed in a range from 0.04 µg to 1.5 µg with a coefficient of determination R^2^ of 0.9999. For the study and characterization of the MM-OPA40, the polyphenol content was expressed as OLE, due to its high amount in the polyphenolic fraction.

### 2.4. Preparation of Micelles (MM) and Polyphhenol-Extract Loaded Micelles (MM-OPA40)

MM and MM-OPA40 were obtained by thin film hydration method [36]. Pluronic F68, Pluronic L121, and TPGS were at a 1:1:0.25 final weight ratio. Two hundred mg of Pluronic L121, 100 mg of TPGS, 200 mg of Pluronic F68, and 150 mg of OPA40 were dissolved in MeOH/CH_2_Cl_2_ blend (8:2), then, the organic solvent was evaporated, and the film was hydrated with 10 mL distilled water for 5 min. Blank micelles were obtained without addition of the extract.

### 2.5. Characterization of Micelles

#### 2.5.1. Particle Size, ζ-Potential, and Morphological Characterization

Droplet sizes and ζ-potential were valued, as an average of three measurements, by using a Zetasizer Pro Red Label dynamic and electrophoretic light scattering (DLS and ELS) (Malvern Instruments, Malvern, UK) at 25 °C. Samples were previously diluted with distilled water and scattering analysis was performed at 90° in a 4 mL borosilicate cell. As far as morphological characterization is concerned, the evaluation of MM’s morphological aspects was achieved by Scanning Electron Microscope Gaia 3 (Tescan s.r.o, Brno, Czech Republic) FIB-SEM (Focused Ion Beam-Scanning Electron Microscope). The electron beam used for the TEM imaging had a voltage of 15 kV, and it was operated in a high-vacuum mode and with a bright-field TEM detector.

#### 2.5.2. Encapsulation Efficiency

The encapsulation efficiency (EE%) of OPA40 was determined through the dialysis method [37]. All samples were analyzed in triplicates via HPLC-DAD analysis. EE% was determined by using following Equation (1):

(1) $$\mathrm{EE}\%=\frac{\mathrm{weight}\;\mathrm{of}\;\mathrm{OLE}\;\mathrm{in}\;\mathrm{micelles}}{\mathrm{weight}\;\mathrm{of}\;\mathrm{OLE}\;\mathrm{used}\;\mathrm{in}\;\mathrm{the}\;\mathrm{formulation}}\;\times \;100\;$$

#### 2.5.3. Cloud Point and Critical Micellar Concentration Determination

For the determination of the cloud point, glass tubes containing 4 mL of MM were put in a water bath at room temperature. Thereupon, the temperature was increased slowly to monitor the change in the dispersion from clear to cloudy. Consequently, micelles were cooled down. The evaluations were performed in triplicate [38].

To determine CMC, non-invasive back scatter (NIBS) optics were used, where light was detected at an angle of 173°, maximizing the detection of scattered light and providing the highest sensitivity [39]. Measurements were made of different concentrations of polymer particles in distilled water and the intensity of scattered light was evaluated by observing autocorrelation functions and kcps (kilo counts per second) values.

### 2.6. Lyophilization

MM and MM-OPA40 were freeze-dried after being frozen under liquid nitrogen. The freeze-drying process took place in a Leybold Heraeus Lyovac GT2 (Leybold GmbH, Cologne, Germany) for the duration of 24 h, the pressure of process was −1.0 bar. After reconstitution of the product with the original volume of water, any changes in terms of mean diameter, ζ-potential, and encapsulation efficiency were evaluated.

### 2.7. Storage Stability of MM-OPA40 and Freeze-Dried MM-OPA40

Storage stability was evaluated for both dispersed micelles and lyophilized formulation over 14 days at 4 °C. Samples were evaluated by visual inspection for changes in aspect, by DLS and ELS for monitor size, PdI, and ζ-Potential, and by HPLC-DAD, to evaluate changes in terms of concentration.

### 2.8. In Vitro Release Studies

The polyphenol release from both the OPA40-saturated solution and MM-OPA40 were evaluated by utilizing regenerated cellulose dialysis membranes with MWCO 12–14 k (Spectrum Laboratories, Inc., Breda, The Netherlands). Two mL of the sample was placed into a dialysis membrane and immersed in 200 mL of PBS with 0.5% Tween 80. Samples were collected at fixed time intervals and the withdrawn medium was replaced with fresh solution. The polyphenolic concentration was determined by HPLC-DAD and the release profile was considered in terms of the percentage of polyphenols released, plotted against time. OPA40 release was also monitored in enzyme-free simulated gastric fluid (SGF) over 2 h and in simulated intestinal fluid (SIF) over a period of 6 h.

### 2.9. Parallel Artificial Membrane Permeability Assay (PAMPA)

For the setup of this in vitro assay, a 96-well MultiScreen-IP filter plate (Millipore corporation) was used to evaluate the ability of OPA40 to permeate from the donor to the acceptor chamber. The acceptor medium contained PBS and the membranes of the filter plates were derivatized with lecithin and cholesterol in 1,7-octadiene. MM-OPA40 or OPA40 solution was then added to the donor chamber, and the filter plates were incubated at 25 °C for 2 h. All samples were centrifugated 14,000× *g*, diluted with MeOH, and analyzed via HPLC-DAD. The permeability coefficient P_e_ (cm/s) was calculated according to the following Equation (2):

(2) $$\mathrm{Pe}=\frac{-\ln[1-C_{A}/C_{\mathrm{eq}}]}{\begin{array}{c} A\left(\frac{1}{V_{D}}-\frac{1}{V_{A}}\right)t \end{array}}$$

C_eq_ (C_equilibrium_) was calculated according to (3):

(3) $$\mathrm{Ceq}=\frac{\left[C_{D}\;\times \;V_{D}+C_{A}\;\times \;V_{A}\right]}{V_{D}+V_{A}}$$

where A is the active surface area (0.3 cm^2^ × apparent porosity of the filter), V_D_ is the donor well volume (mL) and V_A_ is the acceptor well volume (mL), respectively, t is the incubation time (s), and C_A_ and C_D_ are the concentration of polyphenols in the acceptor and donor plate at time t, respectively. The experiments were performed in quadruplicates.

### 2.10. Caco-2 Cell Line

Caco-2 cells are human colorectal adenocarcinoma cells and were purchased from the American Type Culture Collection (ATCC^®^HTB-37™, Manassas, Virginia). Cells were cultured in DMEM medium, supplemented with 2 mM L-glutamine, 100 µg/mL streptomycin, 100 U/mL penicillin, and 10% FBS (complete medium) at 37 °C in a 5% CO_2_-humidified atmosphere. At 80% confluence, cells were detached with 0.025%-EDTA 0.5 mM trypsin solution and propagated after an appropriate dilution.

### 2.11. MTT Assay

The metabolic activity of Caco-2 cells as an indicator of cell viability was investigated via MTT colorimetric assay. Cells were cultured in a 96-well plate (8 × 10^3^ cells/well) in complete medium overnight. Caco-2 cells were then treated for 3 h in complete medium with free OPA40 in the concentration range of 0.52–300 µg/mL, or MM-OPA40 at the corresponding OPA40 concentrations. Then, 100 µL of MTT solution (0.5 mg/mL) was added to each well and incubated for 1 h in the dark. Subsequently, cells were lysed with dimethyl sulfoxide (100 μL/well) and the absorbance values were measured using a microplate reader at 595 nm. The MTT test was repeated in triplicate. The statistical analysis was performed via a one-way analysis of variance (ANOVA) followed by Tukey’s HSD test.

### 2.12. Caco-2 Cell Permeability Assay

In this work, the Caco-2 cell monolayer was used to test the membrane permeability of free OPA40 or OPA40-loaded polymeric micelles (MM-OPA40).

#### 2.12.1. Formation of the Cell Monolayer and the Evaluation of Membrane Integrity

For the cell monolayer formation, Caco-2 cells were plated at a density of 6 × 10^4^ cells/well in a 12-well trans-well plate, using cell culture inserts with PET (polyethylene terephthalate) membranes with pores sized at 0.4 mm and a growth area of 4.5 cm^2^ (Sarstedt, Milan, Italy). Complete medium was added for both the apical (AP) and basolateral (BL) sides of the trans-well filters. The cells were then allowed to differentiate for 15–20 days in DMEM growth medium. The medium was freshly changed every other day in the first week, and daily thereafter.

The integrity of the cell monolayer was verified with the Lucifer Yellow (LY) permeability test [40]. LY was used at a final concentration of 100 µM in a transport buffer (HBSS with Ca^2+^ and Mg^2+^, 25 mM HEPES, pH 7.4) and added to the AP compartment. The transport buffer without LY was added to the BL compartment. After incubation at 37 °C for 1 h, HBSS in the BL chamber was collected and the LY concentration determined using 485 nm excitation and 530 nm emission on Biotek Synergy 1H plate reader (Agilent Technologies, Santa Clara, CA, USA). The percent permeability from apical (AP) to basolateral (BL) was calculated according to Equation (4):

(4) % permeability=(BL fluorescence − blank)/(AP fluorescence − blank) × 100 

The maximum critical flux of LY to identify leaky monolayers was set to be less than 3% of the initial concentration.

#### 2.12.2. Transmembrane Transport Study

For transport studies, the culture medium (DMEM) was replaced with preheated (37 °C) transport buffer, supplemented with 25 mM HEPES (pH 7.4). After that, the cell monolayer was equilibrated for 30 min at 37 °C. Caco-2 cells were then exposed for 3 h with free extract (100–300 µg/mL), or MM-OPA40 at the same concentrations as OPA40, in the AP chamber, while the BL chamber contained only transport buffer. After 3 h, 0.8 mL of medium in the BL side and 2 mL of medium in the AP side were taken for HPLC analyses. At the end of the experiment, the integrity of the layer was re-evaluated with the LY permeability assay as described above. The apparent permeability coefficient (P_app_, cm/s) of polyphenols from free OPA40 and MM-OPA40 was calculated according to Equation (5):

(5) $$P_{\mathrm{app}}=V\_D⁄((A\times M\_D))\times ((∆M\_R)⁄∆t)$$

where V_D_ is the apical (donor) volume (mL), M_D_ is the apical (donor) amount, and ΔM_R_/Δt are the change in amount of compound in receiver compartment over time.

## 3. Results and Discussion

### 3.1. Characterization of the Extract and Preparation of Mixed Micelles

The extract of dried olive leaves (OPA40) is a fraction enriched in oleuropein, with 41.67 g per 100 g of product. The total polyphenols equaled 49.63% *w*/*w*, including, beyond oleuropein, hydroxytyrosol (0.16%), verbascoside (1.18%), oleuroside (4.39%), luteolin (0.02%0, luteolin-7-O-glucoside (1.05%), luteolin-4-O-glucoside (0.46%), luteolin diglucoside (0.06%), apigenin-7-O-glucoside (0.17%), apigenin-7-O-rutenoside (0.13%), 7-epilogalin (0.30%), and eleanolic acid glucoside (0.04%) (Figure S1 of the Supplementary Materials).

This extract was formulated in polymeric micelles, which have a lower CMC, higher kinetic stability, and lower toxicity than micelles made from low molecular weight tensides. Pluronic L121 and TPGS were initially selected as mixed micelles constituents. Pluronics are copolymers that are readily available, biocompatible, with a low toxicity, and are FDA-approved [41]. TPGS was used for its antioxidant properties and its ability to increase the permeation of substances through the intestinal barrier [42]. Blank micelles and OPA40-loaded micelles were prepared with thin lipid hydration method. Initially, blank binary micelles were developed using different weight ratios of Pluronic L121 and TPGS; their physical parameters are reported in Table 1. The selection of the formulation was based on the size and PdI of the system and the characteristics of the micelles after freeze-drying and rehydration. The freeze-drying process was considered to improve the stability of the micelles and the loaded extract. Furthermore, a solid is more easily packaged or transformed into a product, such as capsules and tablets. As evidenced by the results, only the system Pluronic L121/TPGS (4/1) showed good values in terms of dimensions and PdI before the lyophilization and maintained them after the re-dispersion of the solid product. The other weight ratios tested were discarded, due to the instability of the micelles upon lyophilization or the unsatisfactory physical parameters of the colloidal dispersion.

Then, different amounts of OPA40 were loaded into binary micelles (Table 2). The final tested concentrations of OPA40 were 5, 10, 15, and 20 mg/mL. All the samples showed narrow sizes and good homogeneity, with a high EE%. However, only the micelles containing 5 mg/mL of extract maintained the same physico-chemical parameters after the freeze-drying process. Then, the ternary system L121/F68 (1:1)-TPGS (4:1) (MM) was evaluated, to improve the loading capacity and obtain a re-dispersible solid product (Table 3). From the results reported in Table 3, mixed micelles containing 15 mg/mL of extract (MM-OPA40) were selected for further investigations. The MM-OPA40 final composition was as follows: 15 mg/mL of OPA40, 20 mg/mL of Pluronic-L121, 20 mg/mL of Pluronic F68, and 10 mg/mL of TPGS. The recovery percentage was 91.55% ± 4.55 and 87.56% ± 1.88, before and after the freeze-drying.

The hydrodynamic diameter was below 15 nm with a PdI value of 0.19; this is relevant for gastrointestinal uptake as a mean diameter below 100 nm can increase drug absorption. The presence of the extract did not affect the physical and chemical properties of the system. The ζ-potential of the empty micelles was −4.45 mV, and it was −10.22 mV for the MM-OPA40. This, together with the presence of polyethylene glycol in the shell, resulted in the prevention of aggregation, scarce bonding to plasma proteins, and stability in physiological fluids [43]. After the freeze-drying process, no changes in terms of physical properties or EE% were observed (Table 3). The TEM analysis of both MM-OPA40 and the lyophilized product showed sizes comparable to those of DLS and micelles with a spherical shape (Figure S2 of Supplementary Materials).

### 3.2. Determination of the Cloud Point

A cloud point refers to the temperature at which a solution of a surfactant begins to agglomerate, generating a cloudy appearance. The progressive increase in temperature is responsible for polymers’ hydrophilic chain dehydration, which determines the aggregation of the system. The cloud point determination of the optimized system, containing 20 mg/mL of Pluronic-L121, 20 mg/mL of Pluronic F68, and 10 mg/mL of TPGS (MM), was performed in triplicate and the result was 97.33 ± 2.78 °C. The increase in temperature causes the dehydration of the hydrophilic chain of the polymers, resulting in a progressive loss of the stability of the system. In this case, the high cloud point suggests a promising stability of the formulation after oral administration. The high value was due to the presence of Pluronic F68 and TPGS, which have a cloud point of 110 °C [44,45], while Pluronic L121 has a CP of 15 °C [46].

### 3.3. Determination of Critical Micellar Concentration via Light Scattering Techniques

**Figure 1:**
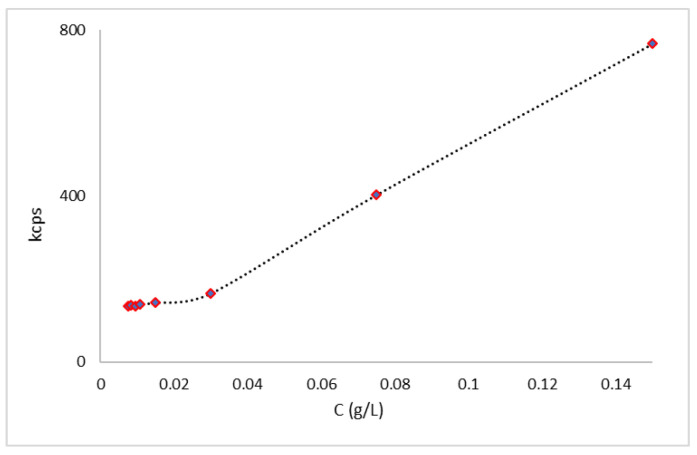
Determination of the CMC of MM using DLS.

The CMC value is defined to characterize the thermodynamic stability of micelles [22,47]. The CMC determination may be carried out through various methods [43]; in this study, the dynamic light scattering technique (DLS) was applied. Measurements were made of different concentrations of polymer particles in distilled water. The micelles’ formation was monitored by the correlation function signal obtained with DLS. Below the CMC, the intensity of scattered light detected from each concentration was similar to that of water, and the autocorrelation functions (kpcs) showed a very poor signal-to-noise ratio. Once the CMC was reached, the intensity of scattered light suddenly increased, due to the self-aggregation of polymers and the formation of micelles; the correlation function allows us to determine the size of the micelles [48]. The CMC value was found to be 3.0 × 10^−2^ g/L (Figure 1). This value is consistent with the CMC values of Pluronic F68 and Pluronic L121 micelles [49,50].

### 3.4. Stability during Storage

**Figure 2:**
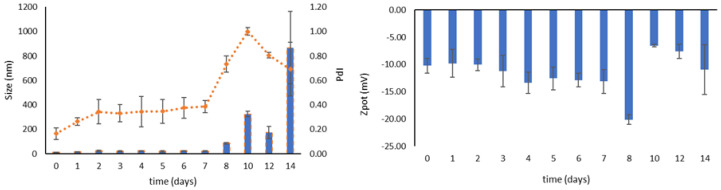
Physical (size, PdI and ζ-potential) stability of MM-OPA40, measured as colloidal dispersion at 4 °C (mean ± SD, *n* = 3).

The physical and chemical stability of MM-OPA40, as colloidal dispersion, was checked over a period of two weeks, to evaluate the micelles’ stability at 4 °C. The MM-OPA40 were stable over a period of seven days, with no significant changes in terms of mean hydrodynamic diameter, and only a slight change in terms of PdI. The instability occurred starting from the eighth day, in particular referring to an increase in the sizes. As far as ζ-potential is concerned, no relevant changes were recorded (Figure 2). The chemical stability over time was evaluated weekly: the EE% decreased to 60.38 ± 4.78% in seven days and reached 57.34 ± 3.65% after 14 days.

**Figure 3:**
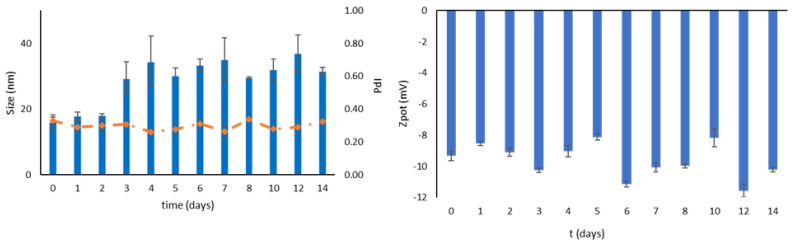
Physical (size, PdI and ζ-potential) stability of MM-OPA40 as a freeze-dried product (mean ± SD, *n* = 3).

After lyophilization, the samples were kept at room temperature in dark conditions for two weeks, to evaluate the enhancement of storage stability in comparison to that of aqueous dispersion. A mild increase in mean hydrodynamic diameter, starting from day three, can be observed, while the PdI value remains constant around 0.3 (Figure 3). The ζ-potential was not affected by freeze-drying as little as EE%, which remained stable over the entire period of the test (from 64.71 ± 2.82% to 61.12 ± 1.18%). Even though a slight increase in terms of size was registered, the lyophilized product remained stable and homogeneous over a longer period of time.

### 3.5. In Vitro Release Study

**Figure 4:**
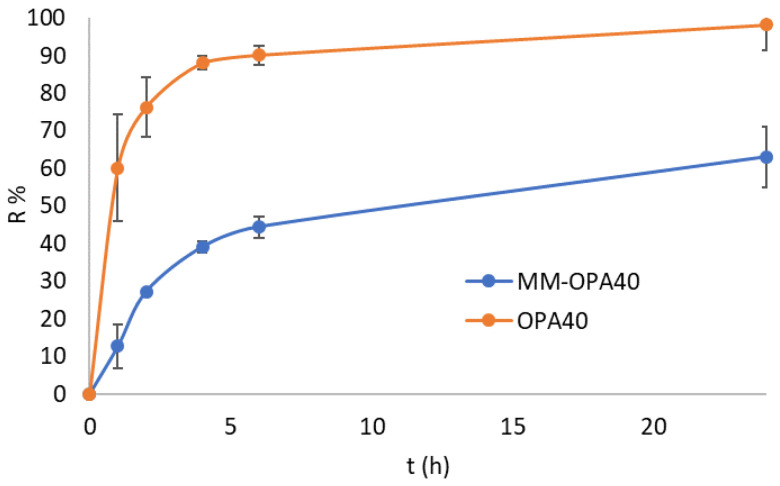
In vitro release of polyphenols from MM-OPA40 and OPA40 solution (mean ± SD, *n* = 3).

The influence of the MM on the release of OPA40 was evaluated with the in vitro release study. The free extract was released from the solution by up to 90% in 4 h and reached 94% after 24 h. Concerning MM-OPA40, the release of the polyphenols was gradual and prolonged; it was up to 46% in 4 h and reached 68% after 24 h (Figure 4). The kinetic of the release was obtained by fitting the release data to various kinetic models and comparing the regression coefficient values (Table 4). The release follows the Higuchi mathematical model and the process is diffusion-controlled, as also reported in other studies [51,52].

**Figure 5:**
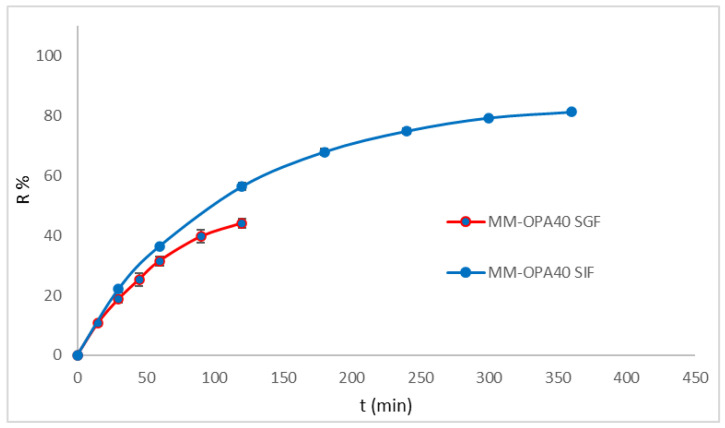
In vitro polyphenol release from MM-OPA40 in simulated gastric fluid and in simulated intestinal fluid (mean ± SD, *n* = 3).

Simulated gastric fluid (SGF) and simulated intestinal fluid (SIF) without enzymes were also considered as release media (Figure 5). Drug release in the SGF was evaluated within 2 h and in SIF within 6 h. The formulation (MM-OPA40) released 40% of the polyphenol in 2 h and 80% in 6 h.

The results proved that MM did not prevent the release of polyphenols. No burst effect was evidenced. The high release rate, particularly in SIF, was related to the composition of MM. As previously observed, Pluronics accelerate the drug release, with respect to other polymers, due to the small micelle size resulting in an increase in specific surface area [53,54,55,56].

### 3.6. Parallel Artificial Membrane Permeability Assay (PAMPA)

**Figure 6:**
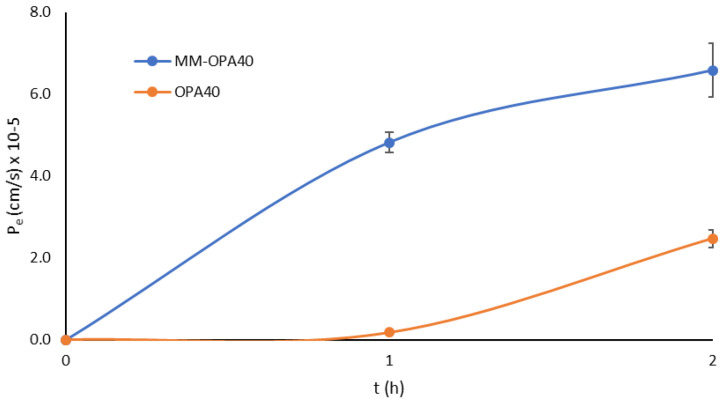
P_e_ (cm/s) values for MM-OPA40 and OPA40 solution (mean ± DS; *n* = 3).

It is well known that log P, together with log D, MW, and ionization, are all key factors in enabling passive uptake. OLE displays a log *p* value of −0.865, with MW of 545, has six hydrogen bond donors and thirteen hydrogen bond acceptors, and, thus, according to Lipiski’s rule, has scarce passive permeability [57]. In the present work, the encapsulation of OPA40 into micelles was intended to protect the extract, but also to enhance passive permeability across the intestinal epithelia. The in vitro PAMPA model is a robust and reproducible model, which is useful for completing the prediction of passive absorption processes by determining the ability of compounds to permeate from the donor chamber to the acceptor chamber. P_e_ was determined for both OPA40 and MM-OPA40, with a significant increase in effective permeability for the formulated extract, as evidenced in Figure 6.

At 1 h, the extract had low permeability (P_e_ 1.74 × 10^−6^ ± 8.90 × 10^−8^ cm/s), which was increased by one order of magnitude by the formulation (P_e_ 4.82 × 10^−5^ ± 2.55 × 10^−6^ cm/s). The same behavior was evidenced at 2 h (with the P_e_ of OPA40 at 2.47 × 10^−5^ ± 2.11 × 10^−6^ cm/s and the P_e_ of MM-OPA40 at 6.58 10^−5^ ± 6.61 10^−6^ cm/s). The recovery was 97%. From the obtained findings, it can be shown that the formulation enhances the passive permeation of the extract across artificial layers. Indeed, TPGS is a nonionic surfactant, widely used as a solubilizing agent, wetting agent, emulsifier, and penetration enhancer [58,59] and Pluronic F68 can also act as a penetration enhancer [60].

### 3.7. Effect of OLE40 and MM-OPA40 on Caco-2 Cell Viability

**Figure 7:**
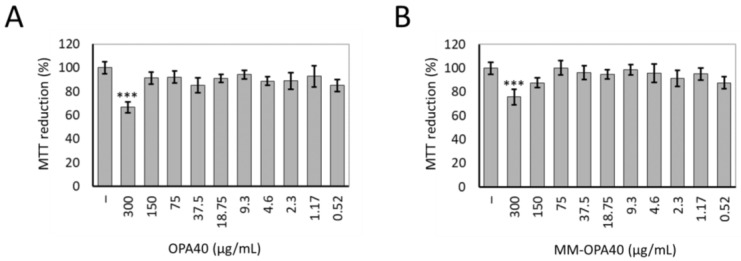
Cell viability of Caco-2 cells treated with OPA40 (**A**) or MM-OPA40 (**B**) in the range of 0.52–300 µg/mL (*w*/*v*) of extract for 3 h. Untreated cells were used as controls (–). The values are given as percentages compared to the control cells. The data were obtained from the mean ± standard deviation of three experiments. Tukey’s HSD test: *** *p* < 0.001 vs. control cells.

The MTT assay was used to determine the viability of Caco-2 human adenocarcinoma cells treated with OPA40 and MM-OPA40, with a range of concentrations of the extract, between 0.52 and 300 µg/mL. It was shown that treatment with OPA40, free or loaded in polymeric micelles, did not result in cell toxicity, up to the concentration of 150 µg/mL of OPA40 (Figure 7). At the highest tested dose of free OPA40 or MM-OPA40, the cellular mortality was about 20%. This reduced cell viability was not attributable to the effect of MM; in fact, cells treated with MM at the corresponding dilution (i.e., 1:50) showed no signs of cell toxicity (Figure S3 of Supplementary Materials). For subsequent transmembrane transport experiments, we chose to work at OPA40 concentrations ranging from 100 to 300 µg/mL.

OLE was poorly absorbed through the intestine by oral administration. As reported in the literature, the apparent permeability coefficient (P_app_) of OLE under iso-osmotic conditions is P_app_ = 1.47 × 10^−6^ cm/s, which categorizes the molecule as a poorly permeable compound [25]. Encapsulation methods and absorption enhancers can represent a promising tool to enhance the absorption of OPA40. The results of this study confirmed the positive effect of micelle composition on the active permeability of polyphenols. After 2 h, the P_app_ was 1.40 ± 0.03 × 10^−6^ cm/s, as reported in the literature for OLE, while the P_app_ of MM-OPA40 was 2.48 ± 0.18 × 10^−6^ cm/s, with a recovery greater than 86%.

## 4. Conclusions

The beneficial effects of various preparations of olive tree leaves have been known since ancient times. Among the secondary metabolites present in olive leaves, there are several polyphenols and, in particular, OLE. In the face of an important biological role, these molecules exhibit poor bioavailability in vivo. In this study, Pluronics–TPGS micelles were developed for the encapsulation and delivery of a polyphenolic-rich extract of an *Olea europaea* L. leaf with high OLE content. The study is part of the objective to valorize olive leaves, which represent a large part of the waste of olive oil production. OPA40 is an olive oil by-product with health-promoting effects and pharmaceutical importance.

Excipients were selected that had good stability and a high loading capacity, while maintaining an absence of cell toxicity. The micelles provided very small droplets, with a small mean diameter, both as aqueous dispersion and as freeze-dried products, and they had a good encapsulation efficiency over a conventional system. In fact, the formulation of an extract into dosage form is a complex process, because the extract contains different chemicals and related compounds that contribute to the pharmacological activity. In this study an EE% of 66% was obtained, corresponding to the 15 mg/mL extract. The lyophilization improved the physical and chemical stability of the polyphenol-rich extract. MM-OPA40 guaranteed a controlled in vitro release of polyphenols and, moreover, a substantial increase in terms of passive permeation, as confirmed by the PAMPA assay and the experiments on the Caco-2 cells. Although further in vivo experiments need to be performed, our new mixed micelles proved to be potential candidates, as a novel formulation, for the delivery of plant extracts, protecting it from instability phenomena and increasing permeation across intestinal epithelia.

## Figures and Tables

**Table 1:** Physical characteristics of binary mixed micelles before and after freeze-drying (mean ± SD, *n* = 3).

Sample	Size (nm) ± SD	PdI ± SD
L121	23.30 ± 1.02	0.25 ± 0.02
freeze-dried product	44.93 ± 1.88	0.58 ± 0.01
L121 + TPGS 4:1	16.09 ± 0.19	0.12 ± 0.05
freeze-dried product	22.60 ± 3.19	0.23 ± 0.09
L121 + TPGS 3:2	14.78 ± 0.27	0.15 ± 0.07
freeze-dried product	60.36 ± 1.55	0.39 ± 0.14
L121 + TPGS 2:3	15.08 ± 0.46	0.29 ± 0.00
freeze-dried product	76.98 ± 1.51	0.32 ± 0.04
L121 + TPGS 1:1	940.5 ± 561.0	0.72 ± 0.25

**Table 2:** Physical (size and PdI) and chemical (EE%) characteristics of L121/TPGS (4:1) binary micelles containing OPA40, before and after freeze-drying (mean ± SD, *n* = 3).

OPA40 (mg/mL)		Size (nm) ± SD	PdI ± SD	EE% ± SD
5	before freeze-drying	15.61 ± 0.05	0.04 ± 0.02	58.81 ± 4.56
	after freeze drying	15.53 ± 0.13	0.06 ± 0.02	61.86 ± 3.28
15	before freeze-drying	15.55 ± 0.11	0.12 ± 0.07	64.91 ± 7.79
	after freeze drying	2166 ± 244	1.00 ± 0.56	32.98 ± 2.61
20	before freeze-drying	15.93 ± 0.03	0.14 ± 0.04	71.18 ± 2.34
	after freeze drying	2356 ± 646.80	1.00 ± 0.79	26.78 ± 3.22

**Table 3:** Physical (size and PdI) and chemical (EE%) characteristics of L121/F68 (1:1)-TPGS (4:1) MM-OPA40 before and after freeze-drying (mean ± SD, *n* = 3).

OPA40 (mg/mL)		Size (nm) ± SD	PdI ± SD	EE% ± SD
0	before freeze-drying	15.13 ± 0.11	0.10 ± 0.01	-
	after freeze drying	16.01 ± 0.18	0.17 ± 0.02	-
10	before freeze-drying	16.36 ± 0.56	0.23 ± 0.03	62.51 ± 1.19
	after freeze drying	14.48 ± 0.12	0.20 ± 0.03	59.89 ± 3.28
15	before freeze-drying	14.21 ± 0.14	0.19 ± 0.05	66.21 ± 1.11
	after freeze drying	15.97 ± 1.13	0.24 ± 0.04	64.71 ± 2.82
20	before freeze-drying	18.22 ± 1.80	0.26 ± 0.03	63.91 ± 6.81
	after freeze drying	2345 ± 222.45	1.07 ± 0.46	39.03 ± 2.52

**Table 4:** Regression coefficient (R^2^) obtained with different kinetic models for OPA40, released from MM-OPA40.

Release Kinetics	MM-OPA40
Zero order	0.6958
First order	0.8270
Korsmeyer–Peppas	0.6812
Hixson	0.7852
Higuchi	0.9144

## Data Availability

The data presented in this study are available on request from the corresponding author.

## Supplementary Materials

The following supporting information can be downloaded at: https://www.mdpi.com/article/10.3390/nano13243147/s1, Figure S1. Chromatographic profiles of OPA40. Figure S2. MM-OPA40 (a) and freeze-dried MM-OPA40 (b) TEM analysis. Figure S3. Cell viability of Caco-2 cells treated with empty MM at various dilutions for 3 h.
