# Supplementary material for: Encapsulation of Olive Leaf Polyphenol-Rich Extract in Polymeric Micelles to Improve Its Intestinal Permeability
Source: Nanomaterials (Basel). 2023 Dec 15;13(24):3147. doi: 10.3390/nano13243147 (PMC10745506; doi:10.3390/nano13243147)
Supplement: Supplementary file 1 [file nanomaterials-13-03147-s001.zip › nanomaterials-2745818-supplementary.pdf]

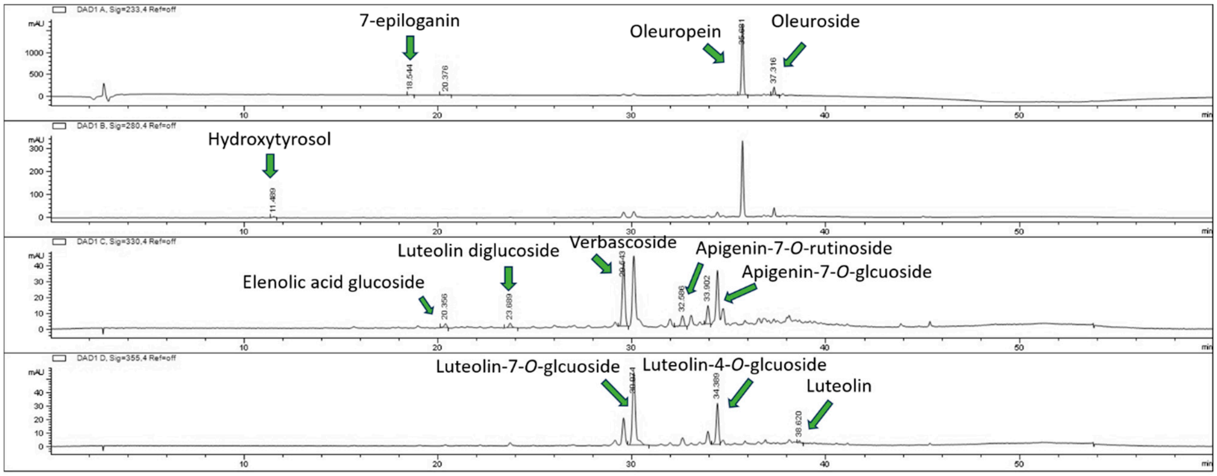

Figure S1. Chromatographic profiles of OPA40.

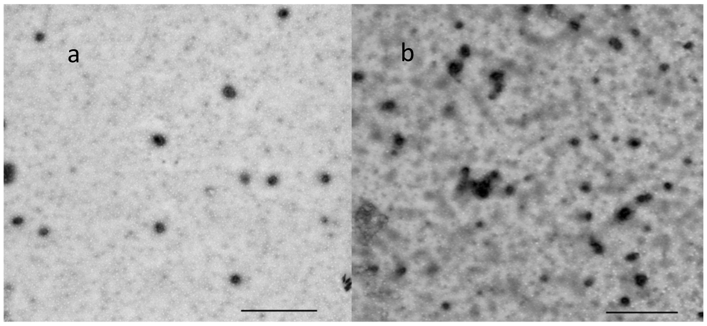

Figure S2. MM-OPA40 (a) and freeze-dried MM-OPA40 (b) TEM analysis. Bar: 100 nm.

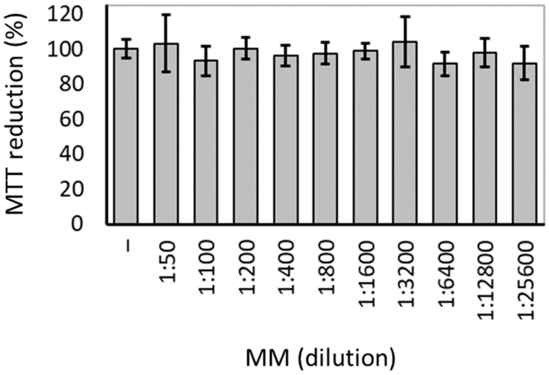

Figure S3. Cell viability of Caco-2 cells treated with empty MM at various dilutions for 3h. Untreated cells were used as control (–). The values were reported as percentages compared to control cells. The data were obtained from the mean ± standard deviation of three experiments.
